# Time-dependent immune and skeletal alterations following total-body irradiation and hematopoietic stem cell transplantation in a murine arthritis model

**DOI:** 10.3389/fimmu.2026.1890654

**Published:** 2026-07-31

**Authors:** Priti Gupta, Riddhi Vyas, Nour Dada, Mattias N.D. Svensson, Marie K. Lagerquist, Carmen Corciulo, Tibor Sághy, Cecilia Engdahl

**Affiliations:** 1 Department of Rheumatology and Inflammation Research, Sahlgrenska Academy, University of Gothenburg, Gothenburg, Sweden; 2 Department of Internal Medicine and Clinical Nutrition, Sahlgrenska Osteoporosis Centre and Centre for Bone and Arthritis Research, Sahlgrenska Academy, University of Gothenburg, Gothenburg, Sweden; 3 Department of Pharmacology, Institute of Neuroscience and Physiology, Sahlgrenska Academy, University of Gothenburg, Gothenburg, Sweden

**Keywords:** bone loss, hematopoietic stem cell transplant (HSCT), immune recovery, osteoimmune imbalance, radiation, local Antigen Induced Arthritis (AIA), inflammation

## Abstract

**Introduction:**

Hematopoietic stem cell transplantation (HSCT) after cytotoxic conditioning, such as total-body irradiation (TBI), is widely used to treat hematologic and immune disorders. However, this treatment is associated with long-term systemic effects, including immune and skeletal complications, and the relationship among irradiation, HSCT, and subsequent inflammatory responses remains poorly understood.

**Methods:**

To investigate the long-term effects of TBI and HSCT on immune and skeletal responses, antigen-induced arthritis (AIA) was induced as a localized inflammatory challenge either 2 weeks (early) or 10 weeks (late) after transplantation. Clinical, immunological, and skeletal outcomes were assessed, including arthritis severity, body weight, immune cell composition, bone morphology, bone turnover markers, and antibody responses.

**Results:**

TBI and HSCT did not alter the severity of antigen-induced arthritis. However, transplanted mice showed greater systemic vulnerability following immune challenge, with reduced body weight, organ atrophy, and increased bone marrow apoptosis, indicating prolonged systemic stress. Bone analyses revealed persistent bone loss after TBI and HSCT, with greater trabecular bone loss in the arthritic limb and elevated serum CTX-1 levels, reflecting enhanced osteoclast-mediated bone resorption. Immune responses also changed over time, with early granulocyte expansion followed by delayed lymphocyte recovery, both associated with increased circulating TGF-β1 levels. In addition, antigen-specific IgG responses were reduced following late immune activation in both naïve and irradiated-transplanted mice.

**Conclusion:**

TBI followed by HSCT induces systemic vulnerability, immune dysregulation, and skeletal deterioration that persists beyond hematopoietic recovery. Although local arthritis severity is unaffected, the host exhibits heightened susceptibility to systemic stress and bone loss after inflammatory challenge, highlighting the long.

## Introduction

Hematopoietic stem cell transplantation (HSCT) is a crucial treatment for hematological cancers and immune disorders, with around 1.5 million procedures performed globally in 2019 (1). Advances in transplantation techniques and supportive care have been shown to have improved long-term survival.

Cytotoxic conditions before HSCT, such as total body irradiation (TBI) and chemotherapy, are essential for successful engraftment of bone marrow cells but also induce long-lasting systemic effects. These effects include structural damage with organ atrophy, chronic immune dysregulation, and impaired skeletal maintenance (2, 3). Additionally, bone tissue is particularly vulnerable to radiation injury because of its high calcium content and the unique metabolic and cellular features of the bone marrow niche (4, 5). Both total body and targeted irradiation reduce bone mineral density, increase fracture risk (6, 7), and ultimately lead to significant morbidity, mortality, and healthcare costs in cancer patients and transplant recipients (8). Recent experimental studies have further demonstrated that radiation-induced bone loss is linked to disruption of the bone marrow microenvironment and multiple osteoimmune mechanisms, including impaired mesenchymal stem cell function (9), cellular senescence (10), hypoxia within the bone microenvironment (11), and Annexin-A1-Dectin-1-mediated osteoclastogenesis (12). Consistent with these findings, we have recently demonstrated that TBI causes bone loss in mice (13). Additionally, we observed that the apoptotic burden in the bone marrow persists for up to 12 weeks post-irradiation and influences osteoclast activity through TGF-β1–dependent pathways.

The immune and skeletal systems are closely interconnected, and disturbances during immune reconstitution may result in a prolonged state of osteoimmune vulnerability. Following cytotoxic conditioning and HSCT, immune reconstitution occurs in a coordinated, time-dependent manner, with innate immune cells recovering first, followed by the slower restoration of adaptive immunity (14, 15). This delayed adaptive immune response increases the risk of infections during the early post-transplant period and may predispose patients to prolonged immune dysregulation. Indeed, autoimmune manifestations involving the hematological, endocrine, neurological, and cutaneous systems have all been reported after HSCT (16, 17). In contrast, it remains unclear whether immune reconstitution also leads to sustained alterations in inflammatory responses and osteoimmune communication that could influence bone homeostasis. The connection between HSCT and inflammatory joint diseases such as rheumatoid arthritis (RA) similarly poorly understood, with only limited case reports describing RA or RA-like syndromes following transplantation (18, 19).

Given the close functional relationship between immune reconstitution and skeletal remodeling (11, 13), cytotoxic treatment and HSCT-induced changes in the bone marrow environment may influence the susceptibility to immune induction and arthritis. To investigate this, we utilized a combined mouse model in which antigen-induced arthritis (AIA), a localized, immune-driven arthritis model, is induced after irradiation and HSCT. By examining early (2 weeks) and later (10 weeks) post-irradiation time points, we evaluated how different phases of immune reconstitution influence arthritis development and related skeletal alterations. Although AIA does not represent spontaneous autoimmunity, it is antigen-specific and depends on immunization with methylated bovine serum albumin (mBSA), which induces local joint inflammation and bone loss (20). Importantly, this model allows direct comparison between inflamed and contralateral joints within the same animals.

Our findings demonstrate that irradiation and HSCT exert time-dependent effects on immune and skeletal responses, identifying a distinct time frame of immune–skeletal vulnerability and subsequent adaptation that may contribute to inflammatory joint pathology after transplantation.

## Materials and methods

### Animals

Seven-week-old female C57BL/6 mice were housed at the Experimental Biomedicine, University of Gothenburg, and fed chow ad libitum under a light/dark cycle. All procedures adhered to ethical guidelines and were approved by the Gothenburg region committees 3230-2020. Arthritis was induced in all mice in two separate experiments: one initiated 2 weeks post-irradiation and the other 10 weeks post-irradiation. These time points were selected to represent early immune reconstitution and later stages of long-term immune recovery following HSCT, allowing assessment of both the acute and persistent effects of irradiation and HSCT on arthritis development. *A priori* power analysis was performed using G*Power version 3.1, indicating that a minimum of 6 mice per group would provide 80% power to detect a 1.5-standard-deviation difference in trabecular bone mineral density between irradiated HSCT and naïve mice (13). Because the study also aimed to evaluate arthritis-related outcomes following irradiation and HSCT and to account for the expected mortality associated with the conditioning regimen, the initial group sizes were increased to 8 mice in the naïve group and 10 mice in the irradiated HSCT groups. The final group sizes ranged from 7 to 9 mice due to mortality following irradiation and HSCT. Both experiments included randomized control groups in which all experimental conditions were identical except for irradiation and HSCT.

### Irradiation and hematopoietic stem cell transplantation

Irradiation and HSCT were performed in a syngeneic transplantation protocol previously established by our group (13, 21, 22). All mice received the antibiotic Enrofloxacin (Elanco ApS), in drinking water one week before irradiation and two weeks after irradiation, to prevent infections in the irradiated mice. Mice were irradiated using the Rad Source model RS2000, equipped with a 0.3 mm copper filter and X-ray tube settings of 160 kV and 25 mA (1.59 Gy/min). A total dose of 9 Gy was delivered in two 4.5 Gy fractions, separated by a four-hour interval.

Hematopoietic stem cells were harvested using the Negative Selection EasySep Mouse Hematopoietic Progenitor Cell Isolation Kit (StemCell) from the femur, tibia, and humerus of female sibling donor mice, as previously described in (13). Cell concentration was measured with a cell counter (Sysmex), and then resuspended in Phosphate-Buffered Saline (PBS). Each irradiated mouse received 500,000 OR 5 x 10^5^ fresh hematopoietic progenitor stem cells in 200 µl of PBS via tail vein injection immediately after the second irradiation, while naive mice received 200 µl of PBS. Based on our previous work, transplantation with as few as 350,000 OR 3.5 x 10^5^ hematopoietic progenitor stem cells has resulted in successful engraftment (22). Mice were weighed before and after HSCT to monitor their health and recovery following irradiation and HSCT.

### Induction of antigen-induced arthritis

Local AIA as previously described in (20, 23, 24), was induced in mice 2 and 10 weeks after irradiation and HSCT. All mice were immunized with 0.1 mg of methylated Bovine serum albumin (mBSA) dissolved in PBS and emulsified with an equal volume of complete Freund’s adjuvant (Sigma-Aldrich). Seven days later, a second injection of 0.015 mg of mBSA in 30 µl of PBS was administered into one knee joint, while the other side received only PBS. The joint swelling, as well as further assessment of arthritis, was measured blindly and recorded daily with a caliper.

Fourteen days after the primary immunization, the mice were anesthetized with ketamine/medetomidine (Pfizer), bled from the axillary vein, and euthanized by cervical dislocation. The total body weight and the weights of the liver, spleen, thymus, and uterus were measured.

### Determination of periarticular bone mineral density using peripheral quantitative computed tomography

To determine periarticular bone loss, the skin and muscle were removed from the leg, and the bone samples were placed in formaldehyde for 2 days, after which they were stored in 70% ethanol. The analysis was conducted blinded bilaterally on both sides using Stratec pQCT XCT Research M (software version 5.4B) (Norland) at a resolution of 70 μm, as previously described (20). Trabecular BMD was assessed in the proximal part of the tibia, in scans performed at a distance of 0.4 mm distal (metaphyseal scan) or proximal (epiphyseal scan) from the growth plate. The inner 45% of the bone area in the sections was regarded as the trabecular bone compartment. Cortical tibial thickness was measured using mid-diaphyseal scans of the tibia. The bone changes between the non-arthritic and arthritic sides were calculated, with no change set to zero and reductions on the arthritic side treated as negative values.

### Histological assessment

The hind legs were decalcified in EDTA with Tris buffer for 3 weeks, dehydrated, and then embedded in paraffin. The knee sections were stained with hematoxylin and eosin (H&E) to measure synovial infiltration and bone destruction. Synovitis, bone erosion, and articular cartilage damage were graded in a blinded manner (by CE and RV) using a three-grade histological scoring system (mild: 1, moderate: 2, or severe: 3) as previously described in (25).

Osteoclasts were identified by tartrate-resistant acid phosphatase (TRAP) staining. Sections were incubated in acetate buffer and TRAP buffer, then counterstained with Fast Green as previously described (23). The tissue sections were mounted on coverslips using Paramount mounting medium (Fisher Scientific). Images were captured under Nikon Eclipse 90i microscope with Nikon NIS-Elements imaging software. Osteoclast number and osteoclast surface-to-bone surface ratio were measured in the epiphyseal region of the tibia using ImageJ software.

The TUNEL assay was performed to detect apoptotic cells using the *In Situ* Cell Death Detection Kit Fluorescein (Roche Diagnostics) according to the manufacturer’s instructions. Briefly, sections were heated and sequentially washed in xylene, ethanol, and water, then treated with Proteinase K in a water bath shaker. Labeling was performed using a prepared enzyme-label solution mix from the kit. Imaging was performed using a Zeiss LSM780 confocal laser scanning microscope with a Zeiss C-Apochromat × 40/1.20 objective (Carl Zeiss) and apoptotic cells in the epiphyseal region of the tibia were observed by using Zeiss Zen 3.1 (blue edition) software.

### Flow cytometric analysis

A single-cell suspension of splenocytes was prepared in PBS by passing the tissue through a cell strainer. The cell pellets were resuspended in a lysis buffer (Tris buffer containing NH_4_Cl) to lyse erythrocytes, then washed in PBS. The total leukocyte count was determined using a cell counter (Sysmex), and cells were incubated with the specific antibody. Anti-mouse antibodies were used for flow cytometry analysis: APC-conjugated anti-F4/80 (eBioscience), PE-conjugated anti-CD19 (eBioscience), APC-Cy5-conjugated anti-Gr-1 (eBioscience), V450-conjugated anti-CD11b (Becton Dickinson), and APC-Cy7-conjugated anti-CD4 (Becton Dickinson). Flow cytometry analyses were performed using FACSVerse (Becton Dickinson) and FlowJo version 10 (FlowJo 10.6.2).

### ELISA serum analyses

Venous blood was collected from the axillary vein in tubes containing a silicon serum separator (Sarstedt). After centrifugation, serum was isolated from whole blood and stored at -80 °C until subsequent analysis. Enzyme-linked immunosorbent assays (ELISAs) to quantify C-terminal type I collagen fragments (CTX-I; Immunodiagnostic Systems), indicative of bone resorption, procollagen type I N pro-peptide (PINP; Immunodiagnostic Systems), indicative of bone formation. Measurement of IL-6 (Invitrogen) and TGF-β1 (Invitrogen) was performed by ELISA, with the latent forms activated prior to detection. The levels of total IgG and its subtypes, IgG1, IgG2b, and IgG3, were quantified using an ELISA (Invitrogen). All assays were performed according to the manufacturer’s protocol. Anti-mBSA-specific IgG was analyzed using ELISA as previously described (23). In brief, plates (Maxisorp, Fisher Scientific) were coated with mBSA, and blocked with 3% casein (Sigma-Aldrich) before adding the serum. Bound anti-mBSA antibodies were detected using HRP-conjugated rabbit anti-mouse IgG (DAKO, Sigma-Aldrich), followed by development of the reaction with TMB substrate.

### Statistical analysis

Statistical analyses were conducted using GraphPad Prism version 10 (GraphPad Software). Normality was checked using the Shapiro-Wilks test. A two-way analysis of variance (two-way ANOVA) was used to evaluate the effects of immune challenge, irradiation, and their interaction across the different time points (2 and 10 weeks post-irradiation). When significant main effects or interactions were detected, Tukey’s multiple comparison test was used for *post hoc* pairwise comparisons. Knee swelling and paired bone differences between the arthritic and non-arthritic sides in mice were analyzed with a two-way ANOVA, followed by Tukey’s multiple comparison. Histological scoring used an ordinal scale, which required nonparametric analysis; therefore, a Mann-Whitney test was performed. Data are presented as bar graphs with standard error of the mean (SEM). A p-value < 0.05 is considered statistically significant.

## Results

### Irradiation and HSCT did not prevent arthritis development

**Figure 1:**
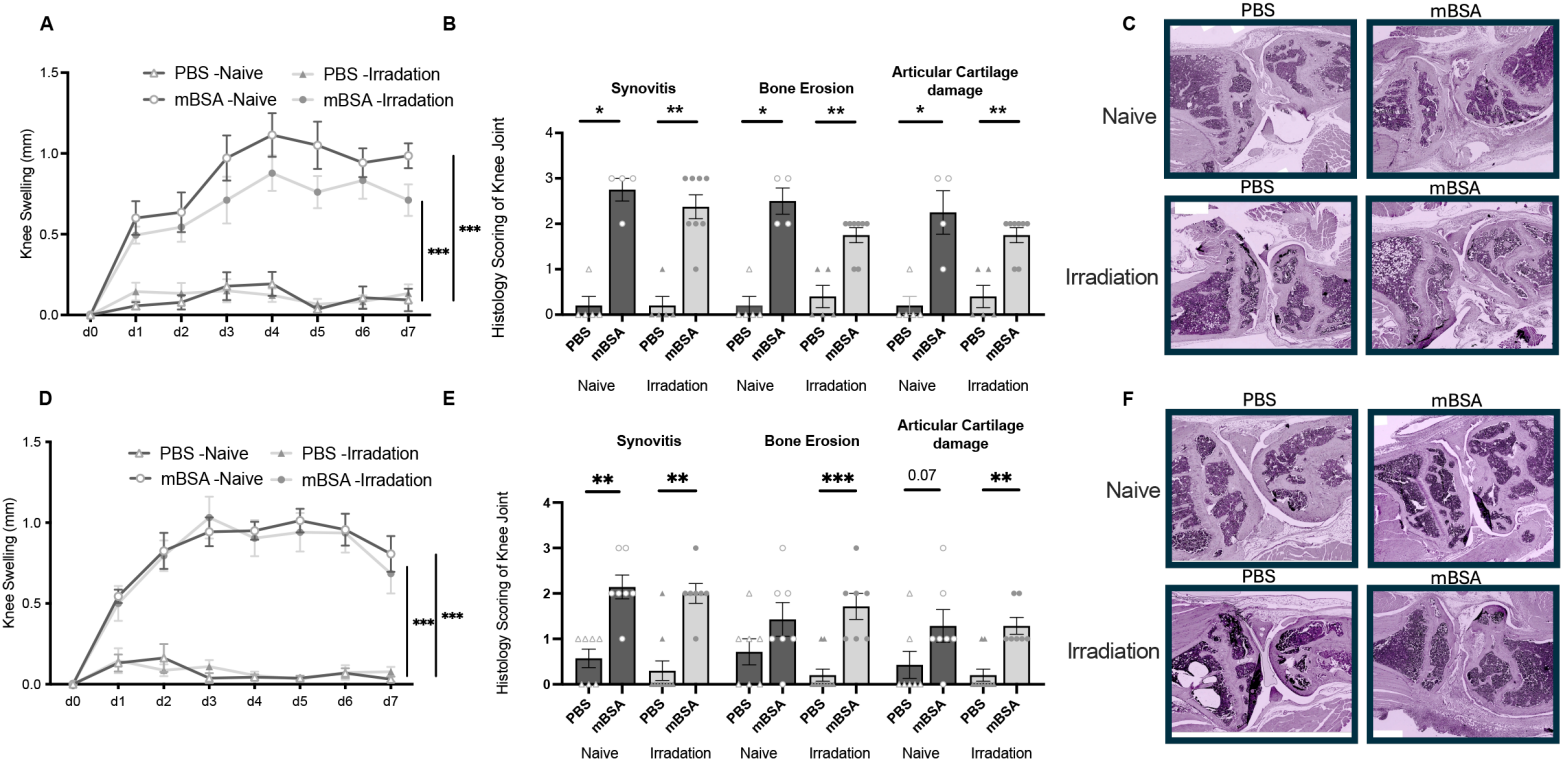
Irradiation does not alter knee swelling or histological signs of arthritis. Early induction of antigen-induced arthritis, 2 weeks post-irradiation: **(A)** Change in knee swelling from baseline (mm). **(B)** Histological scores (0–3) for synovial inflammation, bone erosions, and cartilage damage. **(C)** Representative images of hematoxylin and eosin (H&E) staining of the knee joint. Late induction of antigen-induced arthritis 10 weeks post-irradiation. **(D)** Change in knee swelling from baseline (mm). **(E)** Histological scores (0–3) for synovial inflammation and bone erosions. **(F)** Representative images of H&E-stained knee joints. Data are presented as mean ± SEM. In **A** and **D**, the change was calculated as the area under the curve, and Two-way ANOVA with Tukey's multiple comparisons test was used to test differences between PBS and the mBSA-injected knee. **(B, E)** are ordinal data analyzed with a nonparametric Mann-Whitney test. *N=7-9.* *P < 0.05, **P < 0.01, ***P < 0.001.

To determine whether irradiation and HSCT affected the induction of inflammatory arthritis, mice underwent local antigen-induced arthritis (AIA) following irradiation and HSCT. All mice were immunized with methylated bovine serum albumin (mBSA) and then challenged intra-articularly to induce localized arthritis. Two separate experiments were conducted, using irradiated and HSCT mice from the same round. In the early induction experiment, arthritis was induced 2 weeks after irradiation and HSCT, while in the later induction experiment, it was induced 10 weeks after irradiation and HSCT. The development of arthritis was analyzed separately for each experiment. Data from the 2-week AIA experiment are shown in **Figures 1A–C**, and data from the 10-week AIA experiment are shown in **Figures 1D–F**.

In all mice, intra-articular injection of mBSA into the joint induced arthritis when compared to the contralateral knee injected with PBS, as shown by increased knee swelling and histological signs of synovitis, regardless of irradiation and HSCT (**Figure 1**). Histological examination confirmed synovial inflammation in arthritic mBSA-injected knees of both naïve and irradiated-HSCT mice at all time points (**Figures 1B,C, E, F**). At early 2-week induction of arthritis, mBSA-injected knees exhibited significant bone erosion and cartilage damage compared to PBS-injected control joints in all mice (**Figures 1B,C**). Conversely, at later induction, 10-weeks, mBSA-injected knees in naïve mice showed only a trend toward bone erosion and cartilage destruction, whereas irradiated-HSCT mice developed significant bone erosion and cartilage damage compared with the contralateral PBS-injected joints (**Figures 1E,F**).

### Irradiation and HSCT did not enhance arthritis-induced periarticular bone loss despite increased systemic bone resorption

**Figure 2:**
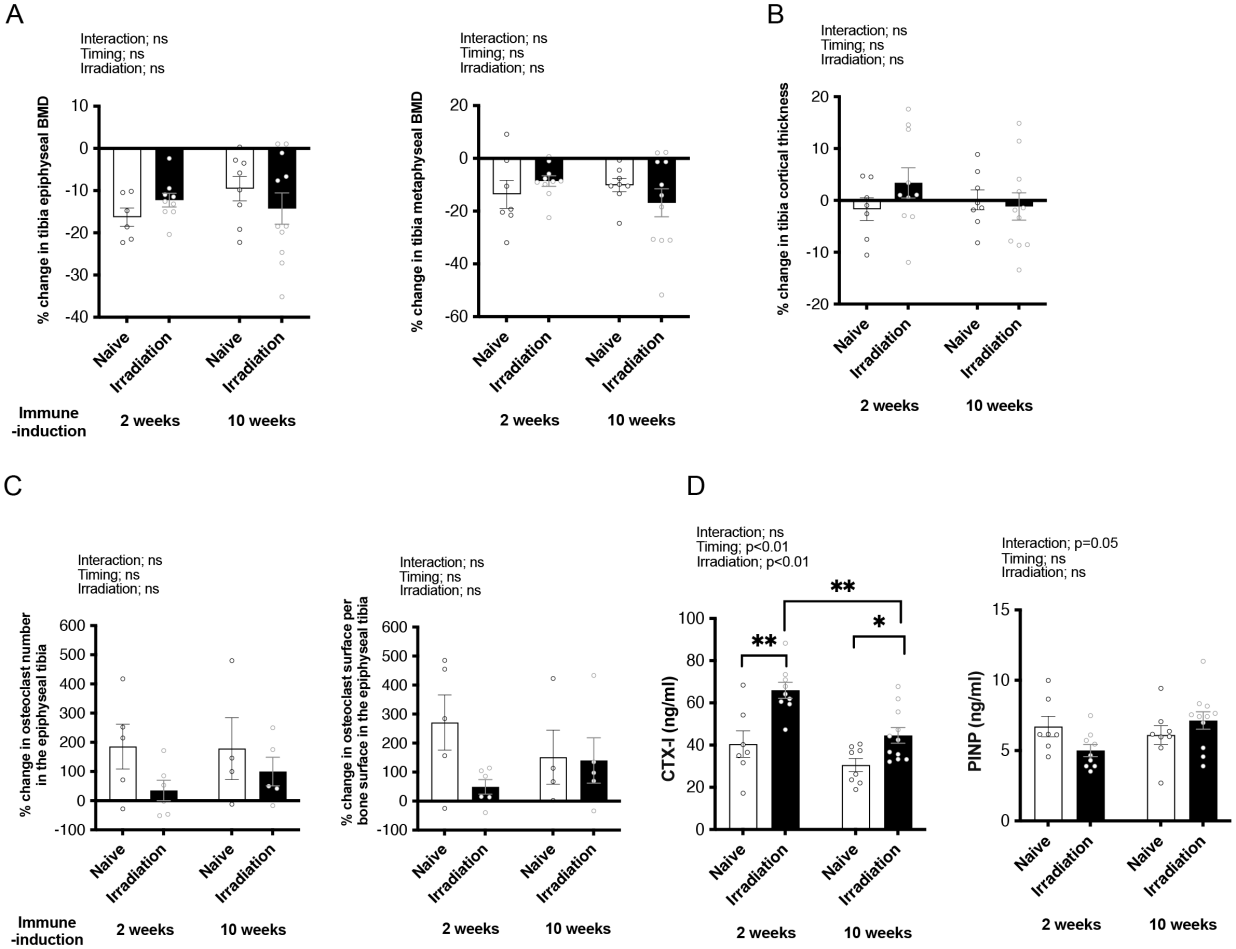
Bone alterations after arthritis induction in irradiated and non-irradiated mice. Changes in bone parameters were assessed as the percentage **(A-C)** change between the arthritic (mBSA-injected) and contralateral (PBS-injected) non-arthritic limbs within the same animals. **(A)** Percentage change in trabecular epiphyseal and metaphyseal bone mineral density (BMD). *N=7-9.* **(B)** Percentage change in cortical thickness. *N = 7-9.* **(C)** Percentage change in osteoclast number and osteoclast surface in the epiphyseal bone. *N=4-7.* **(D)** Absolute number of serum marker levels of bone turnover. C-terminal type I collagen fragments **(CTX-I)** and procollagen type I N-terminal pro-peptide (PINP). Statistical significance was assessed using two-way ANOVA followed by Tukey's multiple comparisons *post hoc* test. Data are presented as mean ± SEM. Significance levels are indicated as *P < 0.05 and **P < 0.01.

To assess the effects of irradiation and HSCT on bone structure after arthritis induction, trabecular bone mineral density (BMD) and cortical thickness were measured in the tibia using peripheral quantitative computed tomography. In both experiments, trabecular BMD in the metaphyseal and epiphyseal regions was significantly lower in the arthritic mBSA-injected side compared to the contralateral side PBS-injected side (**Table 1**). Additionally, irradiation and HSCT further reduced metaphyseal trabecular BMD in the non-arthritic PBS-injected side, but only at the later induction time point (10 weeks) on the arthritic sides relative to naïve mice. No change was detected in epiphyseal BMD post-irradiation and HSCT. However, when bone loss was assessed as the relative difference between arthritic and non-arthritic sides within the same animal, the degree of arthritis-induced trabecular bone loss was similar across all groups, regardless of irradiation-HSCT or the timing of arthritis induction (**Figure 2A**).

**Table 1:** Peripheral quantitative computed tomography (pQCT) bone measurements of tibia bone parameters. .

Bone parameters in Tibia	Early induction, 2-weeks post-irradiation and HSCT				Later induction, 10-weeks post- irradiation and HSCT			
	Naïve		Irradiated-HSCT		Naïve		Irradiated-HSCT	
	PBS	mBSA	PBS	mBSA	PBS	mBSA	PBS	mBSA
Epiphyseal trabecular BMD (mg/cm^3^)	626.9 ( ± 9.9)	542.0 ( ± 21.6) *	637.0 ( ± 24.6)	570.9 ( ± 20.9) *	640.7 ( ± 9.3)	580.6 ( ± 24.2) *	606.8 ( ± 17.9)	532.8 ( ± 25.0) *
Metaphyseal trabecular BMD (mg/cm^3^)	192.4 ( ± 6.6)	164.9 ( ± 7.7) *	178.2 ( ± 10.6) #	164.9 ( ± 9.6) *	194.5 ( ± 13.6)	172.6 ( ± 7.0) *	170.2 ( ± 10.6) #	139.6 ( ± 6.4) *, #
Cortical thickness (um)	19.33 ( ± 0.34)	19.01 ( ± 0.59)	18.23 ( ± 0.58) #	18.73 ( ± 0.41) #	21.08 ( ± 0.27)	21.09 ( ± 0.41)	19.60 ( ± 0.54) #	19.27 ( ± 0.42) #

Bone mineral density (BMD) in the proximal epiphyseal and metaphyseal regions, and cortical thickness in the diaphysis. Data are presented as mean ± SEM. *N=7-9.* animals per group. Statistical significance is indicated by *p<0.05 in a paired t-test comparing the arthritic side (mBSA) to the internal control side (PBS), and # p<0.05 in an unpaired t-test comparing irradiation and HSCT bone to naïve bone. BMD; Bone Mineral Density, mm; millimeter, PBS; phosphate-buffered saline, mBSA; methylated Bovine Serum Albumin, HSCT; hematopoietic stem cell transplantation.

Cortical thickness of the tibia was decreased in both experiments when irradiated-HSCT mice were compared to naïve controls (**Table 1**). However, unlike trabecular bone, cortical thickness was not affected by arthritis induction in either naïve or irradiated-HSCT mice. No alteration was detected when comparing the non-arthritic side with the arthritic side (**Figure 2B**).

Histological assessment of osteoclast parameters showed a tendency toward increased osteoclast number and a higher percentage of the bone surface covered by osteoclasts on the naïve arthritic side compared to the non-arthritic side when arthritis was induced 2 weeks post-irradiation and HSCT, at the early time point (**Table 2**). A similar increasing trend was also observed in the number of osteoclasts in the naïve arthritic side compared to the non-arthritic side when arthritis was induced 10 weeks post-irradiation and HSCT (**Table 2**). In irradiated-HSCT mice, osteoclast numbers were already higher in the non-arthritic PBS side, and no difference was observed between the arthritic and the non-arthritic side. There was a tendency toward higher osteoclast numbers on the non-arthritic side when comparing naïve and irradiated-HSCT mice, in both early and late induction. Relative differences between arthritic and non-arthritic joints showed increased values across all groups (**Figure 2C**). However, the induction was limited in irradiated-HSCT mice.

**Table 2:** Osteoclast parameters in the epiphyseal region of the tibia. .

Osteoclast parameters	Early induction, 2-weeks post-irradiation and HSCT				Later induction, 10-weeks post- irradiation and HSCT			
	Naïve		Irradiated-HSCT		Naïve		Irradiated-HSCT	
	PBS	mBSA	PBS	mBSA	PBS	mBSA	PBS	mBSA
Number of osteoclasts	29.0 ( ± 10.6)	61.4 ( ± 11.4) p=0.12	44.7 ( ± 5.6) p=0.09	54.9 ( ± 12.5)	14.5 ( ± 5.7)	28.0 ( ± 4.4) p=0.14	23.8 ( ± 7.1) p=0.08	27.8 ( ± 5.5)
Osteoclast surface/ Bone surface (%)	5.44 ( ± 2.86)	11.43 ( ± 1.04) p=0.06	9.06 ( ± 1.35)	11.58 ( ± 2.59)	3.03 ( ± 1.77)	5.46 ( ± 0.87)	4.77 ( ± 1.51)	7.46 ( ± 0.58)

Data are presented as mean ± SEM. *N=4-7.* Statistical comparisons were performed using paired t-tests between arthritic (mBSA) and contralateral non-arthritic (PBS) and unpaired t-tests between irradiated-HSCT and naïve groups. A non-significant trend is indicated as p value between p=0.05 and p=0.14. PBS; phosphate-buffered saline, mBSA; methylated Bovine Serum Albumin, HSCT; hematopoietic stem cell transplantation.

Serum markers of bone turnover were measured to supplement the bone assessments reflecting systemic skeletal effects of irradiation rather than local arthritis. Serum levels of C-terminal telopeptide of type I collagen (CTX-I), a marker of bone resorption, were higher in irradiated-HSCT mice compared to naïve mice at both 2-week and 10-week time points after arthritis induction (**Figure 2D**). CTX-I levels decreased over time in all groups, resulting in a smaller difference between irradiated and naïve mice at the 10-week time point. Serum levels of procollagen type I N-terminal propeptide (P1NP), a marker of bone formation, displayed an interaction in which irradiation and HSCT reduced the P1NP levels at the 2-week time point, but increased the P1NP levels at 10-week, although this difference did not reach significance in the multiple comparison analysis.

### Immune cell responses after local arthritis induction in irradiated and HSCT mice

**Figure 3:**
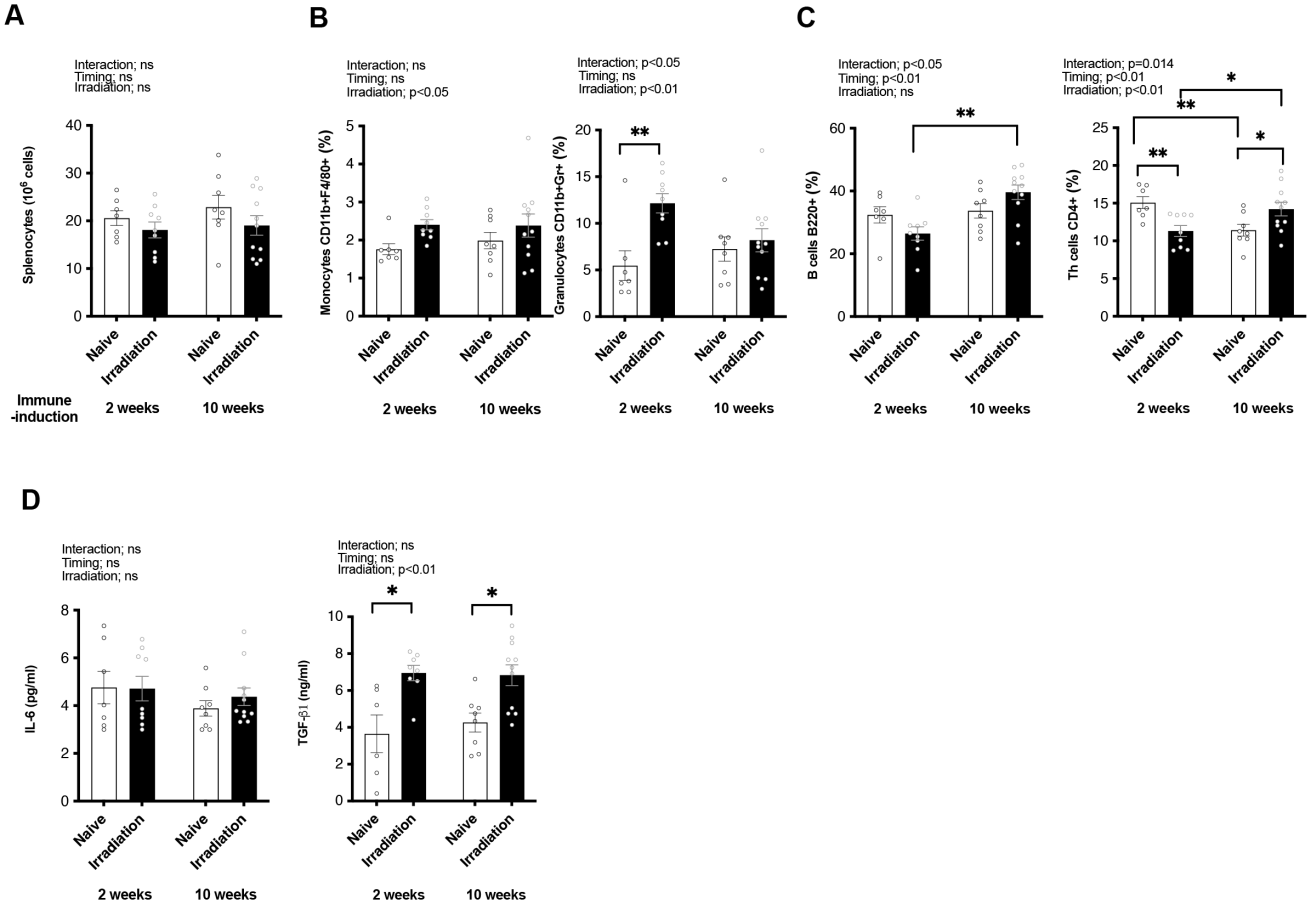
Splenic immune cell composition following irradiation–HSCT and arthritis induction. Effects of irradiation and antigen-induced arthritis on splenocytes. **(A)** Total splenocyte numbers. **(B)** Frequency of myeloid cells, monocytes, and granulocytes. **(C)** Frequency of lymphocytes, B and T helper lymphocytes. **(D)** Serum analysis of IL-6 and TGF-β1 levels. Statistical significance was assessed using two-way ANOVA followed by Tukey's multiple comparisons *post hoc* test. Data are presented as mean ± SEM. *N=7-9.* Significance levels are indicated as *P < 0.05 and **P < 0.01.

Total splenocyte counts remained unchanged by irradiation–HSCT and time (**Figure 3A**). In terms of cell composition, changes were observed. Splenic granulocytes and monocytes increased after irradiation-HSCT in immune-induced mice at both time points (**Figure 3B**). Irradiation and HSCT induced a time-dependent alteration in splenic lymphocytes (**Figure 3C**). In the 2-week, early immune induction, the cell frequencies were significantly reduced in T-helper cells, but not significantly in B cells. This was followed by an increase in the later immune induction 10 weeks post-irradiation and HSCT compared to naïve controls. IL-6 serum levels were not affected by irradiation or HSCT (**Figure 3D**). However, serum TGF-β1 levels were induced by irradiation and HSCT, both at 2 weeks and 10 weeks post-irradiation and HSCT.

### Humoral immune response and antibody production

**Figure 4:**
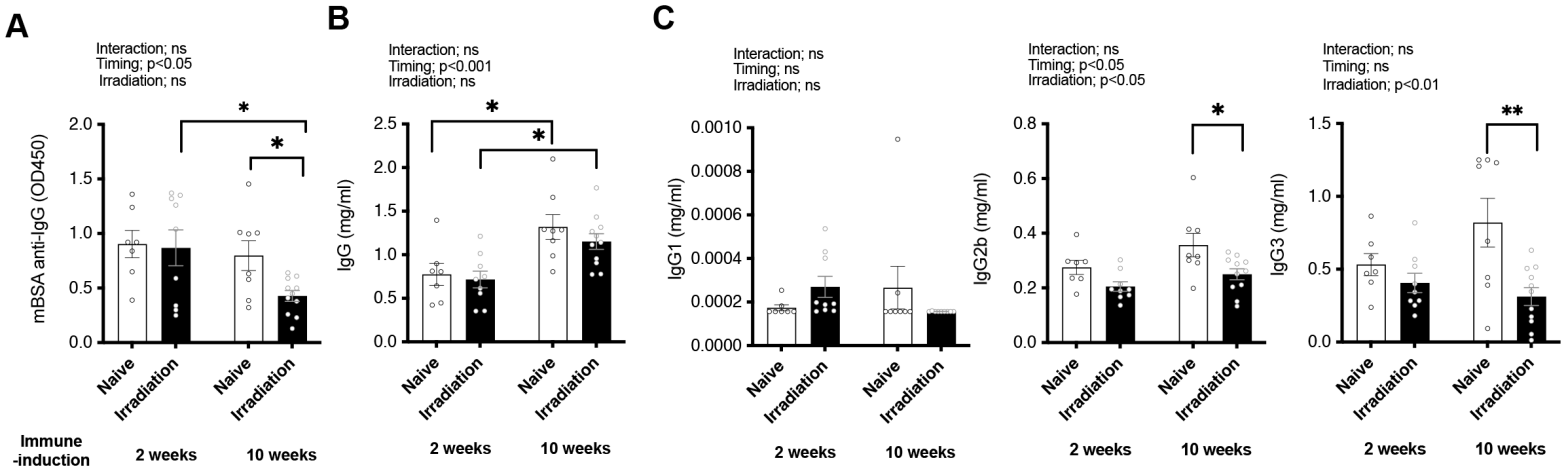
Humoral responses after irradiation–HSCT and AIA induction. Effects of irradiation and antigen-induced arthritis on humoral response. **(A)** Serum mBSA-specific IgG levels. **(B)** Total IgG. **(C)** IgG subclasses (IgG1, IgG2b, and IgG3) were reduced with irradiation after later immune induction. Statistical significance was assessed using two-way ANOVA followed by Tukey's multiple comparisons *post hoc* test. Data are presented as mean ± SEM. *N=7-9.* Significance levels are indicated as *P < 0.05, **P < 0.01, and ***P < 0.001.

All mice produced mBSA-specific IgG after AIA induction with mBSA (**Figure 4A**). These levels were lower in post-irradiation HSCT mice compared to naïve mice after later induction 10 weeks post-irradiation and HSCT. Total IgG levels were increased at the late phase, 10 weeks, compared to 2 weeks post-irradiation, but unaffected by irradiation or HSCT (**Figure 4B**). Subclass analysis showed distinct patterns (**Figure 4C**). IgG1 levels remained stable across all groups. IgG2b levels rose at later induction, 10 weeks in naïve mice, but were reduced in irradiated HSCT mice, while IgG3 levels decreased after irradiation and HSCT, especially in later immune induction, 10 weeks post-irradiation and HSCT.

### Local arthritis exacerbates loss of body weight and uterus, thymus, and spleen weight mediated by irradiation and HSCT

**Figure 5:**
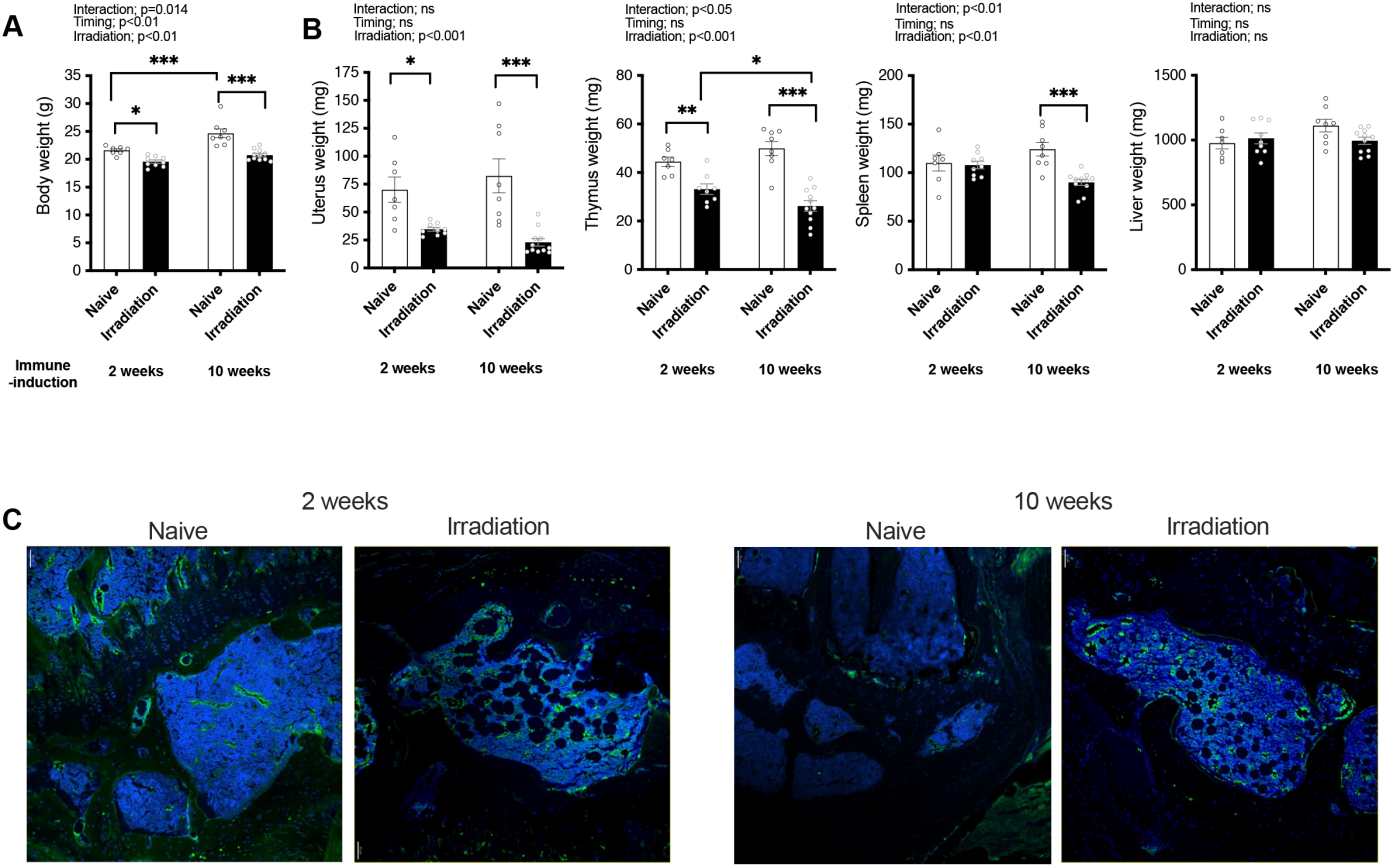
Systemic effects of irradiation and HSCT under immune challenge. **(A)** Changes in body weight in naïve and irradiated–HSCT mice after antigen-induced arthritis (AIA) induction at early (2 weeks) and later (10 weeks) time points post-irradiation. **(B)** Weight of uterus, thymus, spleen, and liver. **(C)** Representative TUNEL-stained femoral bone marrow sections showing apoptotic cells (green) and nuclear counterstaining (blue). All images are acquired at 400x magnification. Statistical significance was assessed using two-way ANOVA followed by Tukey's multiple comparisons *post hoc* test. Data are shown as mean ± SEM. *N=7-9.* Significance levels are indicated as *P < 0.05, **P < 0.01, and ***P < 0.001.

To evaluate the systemic effects of irradiation-HSCT beyond immune and skeletal parameters the weight of the tissue and the mice weight were checked. Total body weight declined immediately after irradiation and HSCT but returned to levels comparable to those of naïve controls within 2 weeks post-irradiation (data not shown). In non-irradiated naive mice, body weight increased with time from 2-week to 10-week induction time points (**Figure 5A**). Indeed, irradiated and HSCT mice showed decreased body weight compared to naïve controls.

Uterus and thymus weights were significantly reduced by irradiation compared to naïve controls, both after arthritis induction and 2 and 10-weeks post-irradiation (**Figure 5B**). Thymic atrophy was more pronounced at the10-week time point. Spleen weight was not altered by irradiation at early immune induction (2 weeks) but was significantly reduced at later time points (10 weeks post-irradiation). Liver weight remained unchanged.

Finally, TUNEL staining revealed increased apoptosis in the bone marrow of the femoral epiphyseal region in irradiated mice compared with naïve mice at both early and late time points (**Figure 5C**).

## Discussion

Total-body irradiation followed by hematopoietic stem cell transplantation (HSCT) significantly disrupts systemic homeostasis and immune composition. In the present study, we show that they do not affect the development of local antigen-induced arthritis (AIA) but induce long-lasting, time-dependent changes in immune and skeletal functions. These alterations include organ atrophy, dysregulated immune cell populations, elevated circulating TGF-β1 levels, defective humoral responses, and elevated trabecular bone loss, as reflected by both serum markers and a tendency toward increased osteoclast number in trabecular bone. Overall, our findings indicate that immune reconstitution after HSCT is functionally reprogrammed rather than fully restored, resulting in persistent systemic vulnerability despite preserved capacity to mount a local inflammatory response.

Despite systemic alterations, local arthritis development occurred similarly in irradiated-HSCT and naïve mice. This shows that the ability to trigger a localized inflammatory response remains functional after immune reconstitution. However, alterations in thymus and spleen weights and in bone tissue indicate that post-transplantation immune recovery compromises tissue resilience rather than initiating inflammation itself. Together, these findings suggest that immune reconstitution restores inflammatory responsiveness without fully re-establishing immune homeostasis.

Consistent with our previous report, we demonstrated that arthritis induced trabecular bone loss irrespective of irradiation (20, 24). Although irradiation-HSCT reduced baseline trabecular bone density, arthritis-induced bone loss relative to the contralateral limb remained similar between groups. Irradiation-HSCT affects skeletal integrity through persistent damage to the bone marrow microenvironment, including apoptosis of resident cells and disruption of the cellular niche required for normal bone remodeling. Subsequent inflammatory arthritis adds immune-mediated bone loss to an already compromised skeleton. Osteoclast numbers tended to increase following arthritis in naïve mice but not after irradiation-HSCT, which may be consistent with a pre-existing osteoclastogenic environment in irradiated animals. However, this interpretation should be approached with caution, as bone erosion and osteoclast numbers were assessed at different anatomical sites, and osteoclast numbers may not fully reflect local bone-erosive activity. Other inflammation-associated mechanisms, including local inflammatory cell-mediated tissue degradation, cartilage damage, and impaired repair or remodeling capacity, may also contribute. Together, these findings are consistent with the concept of osteoimmunology, suggesting that irradiation-HSCT disrupts the bone marrow microenvironment through persistent tissue damage and dysregulated immune reconstitution, leading to altered bone-immune interactions that may influence skeletal remodeling beyond osteoclast activation alone (26). The sustained elevation of CTX-I, prolonged apoptosis, and altered immune composition in the irradiated-HSCT mice further support persistent disruption of bone remodeling and reduce skeletal resilience despite restoration of inflammatory responsiveness.

Immune profiling demonstrated a pattern consistent with post-HSCT reconstitution, characterized by preferential recovery of innate immune cells and delayed restoration of adaptive immunity, consistent with previous reports (14, 27). This pattern supports the concept that functional inflammatory response recovers before complete restoration of immune homeostasis following HSCT.

In all irradiated and HSCT mice, circulating TGF-β1 levels were markedly increased, suggesting the persistent immune modulation after irradiation and HSCT. TGF-β1 is a well-established regulator of immune homeostasis, suppressing effector lymphocyte responses while promoting regulatory pathways and tissue repair (28). Therefore, the elevated TGF-β1 levels observed in this study may reflect an immune reconstitution process skewed toward an immunoregulatory phenotype and tissue remodeling rather than a fully balanced immune response. However, our study findings do not establish a causal role for TGF-β1 in the dysregulated immune phenotype. Similarly, although TGF-β1 has been implicated in regulating osteoclast differentiation and activity (29, 30), this relationship was not directly investigated in our present study. In contrast, serum IL-6 levels remained unchanged, suggesting that the observed immune alterations were not associated with overt systemic inflammation, which could also affect bone. Although the present study was not designed to establish causal mechanisms, our findings support a conceptual model in which irradiation first damages the bone marrow niche, leading to prolonged apoptosis and altered immune reconstitution. Preferential recovery of innate immune cells, delayed restoration of adaptive immunity, and sustained elevation of TGF-β1 may collectively reshape the osteoimmune niche, promote bone remodeling while preserving the capacity to initiate local inflammatory responses. This framework highlights how radiation-induced changes in the bone marrow microenvironment may have long-lasting consequences for immune and skeletal homeostasis after HSCT.

Humoral responses were selectively impaired. Total IgG levels increased over time, likely reflecting age-related maturation of the humoral response, as we previously demonstrated (23). Antigen mBSA-specific IgG responses were reduced following later immune activation in irradiated-HSCT mice. Additionally, IgG2b and IgG3 subclasses were decreased, indicating qualitative changes in adaptive immune function. These findings are consistent with prior work showing that conditioning regimens impair B-cell maturation, reduce antibody repertoire diversity, and disrupt germinal center responses, thereby limiting effective humoral immunity after HSCT (27, 31). The continued production of antigen-specific IgG, although diminished, suggests some functional recovery and also reveals limitations in adaptive immune competence later after irradiation and HSCT.

Irradiation-HSCT also induced systemic structural damage, including thymic and uterine atrophy and sustained bone marrow apoptosis, consistent with previous reports of long-lasting alterations in the bone marrow microenvironment following conditioning regimens (11, 13). Notably, body weight recovered prior to arthritis induction but declined again after immune activation, further supporting incomplete physiological recovery despite the restoration of inflammatory responsiveness. Thymic atrophy is consistent with prolonged impairment of thymic-dependent immune reconstitution after HSCT (32).

Several limitations should be acknowledged. Intermediate time points were not assessed, limiting insight into the kinetics of immune and skeletal recovery. In addition, histological assessment of synovitis was performed, but local immune profiling within arthritic joints and bone marrow were not, which may have provided further mechanistic insight. The antigen-induced model also has limitations, as it is not a spontaneous autoimmune arthritis model. However, it allows direct comparison between the arthritic and non-arthritic joints within the same animal, thereby reducing inter-animal variability. Furthermore, because all irradiated animals received HSCT, the present study cannot further distinguish the individual contributions of total-body irradiation and HSCT to the observed immune and skeletal alterations. Consequently, the findings should be interpreted as reflecting the combined effects of irradiation and hematopoietic reconstitution. Finally, osteoclast quantification was performed in only a subset of animals, which may have limited the statistical power to detect differences and should be considered when interpreting the relationship between osteoclast numbers and bone erosion. Nevertheless, the present model captures key post-irradiation and HSCT features—including immune imbalance and bone fragility—and provides a useful platform for studying therapeutic strategies to restore immune–skeletal homeostasis.

Clinically, these findings suggest that HSCT survivors may retain the capacity to mount inflammatory responses while experiencing compromised immune balance and reduced skeletal resilience, depending on both structural damage and the inflammatory response. Inflammatory arthritis developing in an already osteopenic skeleton may have greater structural consequences, even with similar inflammatory activity. This concept is consistent with clinical observations that osteoporosis exacerbates the consequences of inflammatory bone loss in rheumatoid arthritis (33). Early monitoring of bone density and targeted anti-resorptive interventions may therefore be beneficial in post-transplant management.

In summary, total body irradiation followed by HSCT induces a persistent osteoimmune remodeling characterized by dysregulated immune reconstitution, elevated TGF-β1 signaling, and enhanced bone resorption. Although local inflammatory responses remain intact, systemic immune and skeletal vulnerability persists. These findings highlight the importance of coordinated immune and bone health management in long-term HSCT survivors.

## Data Availability

The data presented in the study are deposited in the Figshare repository, accession number 10.6084/m9.figshare.33078158.
